# Medical Items and News of Interest to the Physician

**Published:** 1874-01

**Authors:** 


### For the Use of Physicians.

CULLED FROM THE STANDARD MEDICAL JOURNALS OF THE WORLD.

NOTE.—These formulæ are intended only for the regular practitioner of medicine, and should be employed only by him, or under his immediate supervision.

**Chancroids.**

First a free cauterization with nitrate of silver or nitric acid, and after the separation of the slough, dress the ulcer with iodiform. The results attending this method of treatment are uniformly good.—*Record.*

**Spasmodic Asthma.**

Nitrite of amyl is considered very good but the prescription ordinarily used is a drachm of cp. spts. of ether with ten minims of tr. of belladonna three times a day. This mixture may be continued for weeks, and many patients improve markedly under its influence.

**Ulcers.**

The dressing which gives the most satisfactory results for cleansing a foul, sloughy, ulcerated surface, preparatory to strapping, consists of a solution of chloride of lime, one half ounce to a pint of water, and applied three times a day upon a cloth or pledget of lint.

**Glycerine in Subcutaneous Injections.**

Dr. Constantin Paul recommends glycerine as a dissolvent for subcutaneous injections. He considers it to be far superior to water, alcohol, etc.; it is neutral, can be kept easily, and is, of all liquids, the one which approaches the nearest to the composition of subcutaneous cellular tissue.—*Jour. Chem.*

**Influence of Belladonna on Sweating.**

In some interesting communications to *The Practitioner*, Dr. Sidney Ringer brings forward an abundance of evidence to prove that belladonna and its active principle are able to check and prevent sweating, whether the result of disease or induced by exposure to an elevated temperature. In the former case, his observations enabled him to conclude that one two-hundreth of a grain of atropia injected under the skin is generally sufficient to check sweating for one night. This dose produces a dryness of the fauces, but does not dilate the pupils. Stramonium, it was found, is able to exert the same influence.

**Delirium Tremens.**

The standard prescription for this condition, in the Roosevelt Hospital, N. Y., is—

℞

Chloral hydrat., grs. xxx;
Potass. bromid., grs. lx. M.

To be given at bedtime, and continued through the day in small dozes if necessary.

**Gastro-enteritis.**

Some cases of this character, which were characterized by loose passages of the bowels from two to four in number daily, and more or less of gastric disturbance, have, after resisting other remedial measures employed, rapidly improved by use of a pill composed of nitrate of silver, ¼ of a grain, and sulphate of morphine, ⅛ of a grain, three times a day.

**Phosphorus Mixture.**

Dissolve a grain of phosphorus in six drachms of chloroform, and add two ounces of good glycerine, and shake well. The glycerine forms a permanent union with chloroform, which dilution with water does not separate. Dose, a teaspoonful three times a day. This is a very pleasant preparation of phosphorus, in the fluid form.—*Boston Jour. Chem.*

**Phthisis.**

The administration of oxygen gas has been commenced in a few cases of phthisic. Two cases which were receiving the oxygen were in the third stage of the disease, both having cavities in their lungs. So far as tried, the effects have been very pleasant and exceedingly gratifying to the patients. The tonic effect realized has been very marked. The patients have slept better, and the appetite has been much improved. The amount administered was two gallons twice a day.

**Abscesses.**

The popular method of removing pus from the cavity of an abscess is by means of the aspirator. As means of diagnosis, or a means for the simple removal of the fluid from a cavity, it is one of the most successful and convenient. As a curative measure it is not regarded so favorably. The simple removal of the fluid in many cases is not sufficient to prevent its return, and it becomes necessary to lay the walls open by a free incision; secure a drainage by seton and cause the cavity to heal from the bottom.

**Chloral with Cod-Liver Oil.**

An Italian medical journal recommends the addition of chloral hydrate to cod-liver oil. It renders it much less nauseous, and prevents the night-sweats of the phthisical patient, induces sleep, and creates appetite. The pure chloral-hydrate crystals may be added to cod-liver oil in the proportion of ten grains of the former to one hundred and ninety of the latter.

---

**Spermatorrhœa.**

℞

Ferri sulph.,

Acid, nitric, āā, ʒ j ;

Aquæ ℥ j ;

Quiniæ sulph., ʒ ss ;

Acid, arsenic, et

Strychnia sulph., āā gr. j. M.

Sig. gtts. x, t. i. d.

The above prescription is regarded as more than ordinarily efficacious.--*Med. Record.*

---

**Inflammability of Ether.**

In a late number of the *British Medical Journal*, Mr. Hutchinson, writing upon ether, expresses an apprehension of danger from the inflammability of the vapor, and advises against its use after dark. Ether is employed at our hospitals indifferently by day and night—as it also is in midwifery. Its practical safety is doubtless partly owing to the fact that the air, cooled by its evaporation, establishes a downward current, so that a match placed a few inches above an ether sponge at the edge of a table will not ignite it, while below the vapor readily takes fire.

---

**Acute and Subacute Rheumatism.**

The leading feature in the treatment of these cases consists in the administration of iodide of potassium in doses varying from 8 to 15 grains, combined with 15 drops of the wine of colchicum-seeds, three times a day. To relieve the local pain about the joints, the application of ice in bags has proven most satisfactory. When the acute symptoms have been subdued, or have subsided, the application of kerosene oil to the stiffened joints, accompanied with free rubbing of the parts, has afforded more relief than other applications. Whether the virtue is in the oil, or in the rubbing, or in both, can hardly be stated, but the beneficial results which have been achieved from its use warrant its adoption.

**Epilepsy.**

A somewhat remarkable case of this disease was under treatment, in which the fits made their appearance within fifteen minutes after receiving a fall and striking upon the head. There were no other symptoms present in the case except those relating to epilepsy. The fits, upon admission of the patient, were exceedingly frequent; but under the administration of bromide of potassium, in large doses, the number and severity of the attacks have very much diminished, and the patient is feeling very well.

---

**Facial Erysipelas.**

Prof. D. M. Salazar, of the Hospital Nacional, Madrid, reports, *El Amfit. Anat. Espan.*, that he had cured eight cases of facial erysipelas in forty-eight hours by glycerine of borax.—He applied the solution to the diseased parts with a brush, and then covered them with a mask of raw cotton. After twenty-four hours all the symptoms, local and general, were notably diminished, and the next day all the phlyctenulæ had disappeared and desquamation was commencing. All medication was then discontiued, except that a decoction of sambucus and althæa was used as a wash to favor the desquamation.—*Med. and Surg. Reporter.*

---

**Pertussis.**

Dr. Thomas S. Davis gives in the *Medical Times* (Dec. 28, 1872) the results of the treatment of fifteen cases with fluid extract of castanea. The first eleven had the characteristic whoop, the remainder had well-marked paroxysms, but not the full spasm, and they recovered without having it. In each case the violence of the spasms was reduced even more markedly than the number of the paroxysms. The castanea was continued for a week, after which, in a few cases, a simple expectorant was given. The nurse in charge, who had witnessed many epidemics of the disease, declared she had never seen medicine act like it.

This medicine is made from the beans of the common chestnut tree, castanea vesca, natural order *Cupuliferæ.* The preparation used was the fluid extract made by Mr. John M. Maisch, of Philadelphia (see *Amer. Journ. of Pharmacy*, Dec., 1871, p. 529). The dose is half a teaspoonful to a teaspoonful every three or four hours, for a child six years old.

**Picric Acid as a Test for Albumen.**

M. Galippe recomends, as an extremely sensitive and thoroughly reliable test for albumen, a saturated solution of picric acid. In normal urine, this solution never causes precipitation, while the most minute traces of albumen are readily detected by it. A drachm or two of the picric acid solution is put into a test-tube, and a few drops of the fluid to be tested are allowed to fall into the solution. If albumen is present, it traces a characteristic white line through the solution.—*Boston Journ. Chem.*

---

**Perchloride of Iron.**

Dr. H. L. Snow remarks that delicate patients very frequently object to the astringent metallic taste long remaining in the mouth after the administration of tincture of perchloride of iron, the flavor of which is but imperfectly disguised by the syrup or spiritus chloroformi with which it is usually ordered. It may not, perhaps, be too insignificant a matter for mention, that the substitution of a small qantity of glycerine (about half an ounce to an eight-ounce mixture) will altogether obviate this inconvenience.

---

**Hemi-Chorea.**

A case of this rather unusual form of the disease was noticed, affecting the right side, and was directly traceable to fright as its cause. A variety of treatment had been instituted in the management of the case, but it yielded only to the administration of carbonate of iron in free doses. The patient was a young girl, and although it may be questionable whether the subsidence of the disease was due to the remedy administered or to the natural progress of the disease, yet it is but fair to state that there was no amelioration in symptoms previous to the administration of the iron, and that improvement immediately followed.

---

**Chinese Treatment of Tetanus.**

This mode of treatment of tetanus has been seen by English physicians in China and India to be successful: The patient smokes in a pipe a mixture of from twenty to twenty-five centigrammes, of crude opium and tea or rose leaves, which are worked up with a small quantity of molasses. When smoking, he must inspire as deeply as possible, and continue this operation until the narcotic influence is noticed. This continues then, as a rule, three or four hours. The smoking is repeated as soon as the tetanic symptoms reappear. In the meantime as much nourishment as possible is given. In using opium thus, it must be remembered that its narcotic effect is somewhat neutralized by tobacco.

---

**Granular Lids.**

A number of cases have been successfully treated in the Roosevelt Hospital, by the use of liq. plumbi subacetatis, after all ordinary means has failed. In one case ectropium was present in a marked degree and with this treatment had almost entirely disappeared. About two drops of the undiluted liquor are dropped into the eye, once in three or four days. It is important that the remedy should not be applied too often, lest the irritation produced should be continued, and prevent the beneficial effects which may follow if used at somewhat lengthy intervals. Give the lids an opportunity to get better after each application. —*Med. Record.*

---

**Eczema Capitis.**

Cure it, if possible, notwithstanding the somewhat prevalent idea to the contrary.

First, apply a poultice every night until all the scabs are removed. The ulcerations, which are sometimes present after the scabs have been removed, are best cured by the application of a wash made of nitrate of silver, grs. v. to the ℥ j. of water. The following is then used with good success:

℞

Aquæ cologn, ℥ iv;

Glycerine, ℥ ij;

Carbolic acid, crystals, ʒ j;

Borax, ʒ j. M.

Continue this application until the disease is cured.

---

**Hydrophobia.**

In a late number of the *Correspondenz-Blatt*, Dr. Guisan gives a number of cases showing the value of arsenic as a prophylactic in hydrophobia, and even as a remedy also after the symptoms are marked. He relates that a rabid dog, between the 7th and 9th of June, bit thirteen persons in various towns of the canton of Freiburg. All were recommended to be treated with one-twentieth of a grain of arsenic morning and evening, as a prophylactic measure. Eight submitted to this prophylactic measure, and none were affected. Four declined, or were not allowed to take the arsenic. Of those four, two remained unaffected, and two died. One began the arsenic treatment, but speedily left it off; she was attacked, but at a much later period, and died. Dr. Guisan recommends not only the internal employment of the arsenic, but that the wound should be dressed with it.

**Green Coffee in Gout.**

Dr. Monchaux, says *The Doctor*, communicates to *L'Abeille Medicale* his experience in the use of cold infusion of green coffee in the treatment of gout. He directs his patients to take a tablespoonful of green coffee, place it in a tumbler, half fill the latter with water at the temperature of the atmosphere, and allow it to stand for twenty-four hours. In the morning the liquid is to be swallowed, an equal quantity of water added, and taken the following morning; thus the same quantity of coffee will serve for two doses. The liquid thus obtained has little savor; it is more or less distinctly green, according to the species of coffee employed. Dr. Monchaux does not explain the mode of action of the remedy upon the uric diathesis. He inclines to the belief that it acts against the effects of the disease rather than on the disease itself, and that, in order to prevent a recurrence of gout, it is only necessary to make a daily use of the remedy indicated by him. He observes that the remedy is an old one, and that, however difficult it may be to explain the precise action, the efficiency of the remedy is acknowledged. He had himself used it three years, and with the result that his bi-annual attacks of gout had completely disappeared.

**Application for Chilblains.**

F. Rhien recommends an aqueous solution of iodine and tannin as a remedy for chilblains. He says that the remedy exceeded his expectations, five applications of the remedy being successful. The application has also been tried by others, with good results when properly applied. The solution is made as follows: About an ounce of tannin is dissolved in half a pint of water; seventy-four grains of iodine are dissolved in an ounce and three-fourths of spirits of wine; the two solutions are then mixed, and enough water is added to make up the whole to two and a half pints. The remedy is applied once daily, the best time being before going to bed. The mixture is gently warmed over a very slow fire; the affected part (*e. g.*, the hand) is dipped in it while still cold, and held there until the liquid, on being stirred, feels uncomfortably hot. The vessel is then removed from the fire, and the hand is dried over it, without gloves. The vessel used must be of earthenware or porcelain, not of metal. Care should be taken not to use too great a quantity of iodine, especially when abrasions are present. According to Rhien, four or five applications are sufficient.—*Apotheker Zeitung.*

**Oxide of Zinc in Infantile Diarrhœa.**

In the *British Medical Journal* of July 12, Mr. Edward Mackey, M. B., recommends oxide of zinc in infantile diarrhœa, especially in those forms complicating nerve-troubles or whooping-cough. He points out that it has "tonic and anti-spasmodic as well as astringent qualities, a combination in a non-irritant substance exactly suited to many cases of the malady." It does not irritate, as chalk sometimes does, or gripe, as acids do occasionally, and is a better nerve-tonic than bismuth. Mr. Mackey says that it has given him excellent results in all varieties of infantile diarrhœa, in conjunction with suitable diet. Dose, one grain for any age under two years, with a little syrup, mucilage and dill-water three or four times daily, not on an empty stomach. A larger dose may nauseate. He says that oxide of zinc will give us in many cases the maximum of good with least liability to harm,—an advantage to be desired, especially in out-patient and distant cases. Dr. Waring-Curran has referred to this use of oxide of zinc, in the *Lancet*, October, 1868, and Dr. Brakenridge, in the *Medical Times and Gazette*, February, 1873, points out its tonic and anti-spasmodic as well as astringent properties.

**Simple Method of Skin Grafting.**

A recent number of the *British Medical Journal* contains an account of Mr. J. Bell's method of skin grafting, practised at the Royal Infirmary, in Edinburgh:

In procuring portions of skin for grafting, Mr. Bell takes them from some sound portion of the patient's body, preferably from the arm. A piece of skin is pinched up by a pair of common catch-forceps, and cut off, to the required size with a pair of scissors. The piece is divided into smaller pieces about the size of a grain of rice, and is planted among the granulations of the ulcer by means of a probe, one small piece being sufficient for about a square inch of surface. Over each of the grafts is laid a piece of gutta percha tissue, half a square inch in size, previously dipped in some antiseptic solution. The ulcer is then covered by two layers of similar pieces of gutta percha tissue placed on each other in an imbricated manner, and over these a dressing of antiseptic gauze and a bandage. This dressing is not removed for two or three days, when it is replaced as at first. To insure success before grafting, the ulcers should be free from fœtor, and the dressing changed under spray. The advantages of this method of grafting are alleged to be, that no special apparatus is required ; that it is extremely simple ; and that, by the movement of the pieces of gutta percha on each other, the grafts are protected from all sources of disturbance.

---

**Empyema.**

The practice of making a free opening through the chest walls in these cases, drawing off the pus, and washing out the pleural cavity, has been adopted in some cases in this hospital. In one case 108 ounces of pus were removed in this manner, and in another 60 ounces. Of course their constitutional treatment was pre-eminently supporting. These patients at present are in good condition, and steadily improving. In the same ward were other patients who had empyema, and had been treated by removing the fluid in the usual manner, and with the same general constitutional treatment as was adopted in the first mentioned cases; they were in good condition and steadily improving. The amount of fluid in the pleural cavity in these cases, however, was far less than in those in whom the free incisions were made ; and the question with reference to the treatment is, would those patients who had such an immense amount of pus in the pleural cavity, have done equally as well if they had been treated after the ordinary method by simple puncture, as they have done by making a free opening into the chest, and having the cavity daily cleaned by some slightly stimulating and disinfecting fluid ? Carbolized water, varying in strength from 5 to 15 per cent., according to the susceptibilities of the patient, is the fluid ordinarily employed for the irrigation of these cavities.

**Veratria in Heart Disease.**

Dr. Bitot, of Paris, lately read a paper on the employment of veratrine in cardio-vascular affections not yet arrived at the period of cachexia. In consequence of experiments in the laboratory, and clinical observations, M. Bitot has been able to determine what is the mode of action of veratrine ; and comparing these results with similar ones obtained with digitalis, he has deduced the following conclusions :

Veratrine is a precious agent for vascular troubles, more especially for those troubles which accompany functional hypertrophy of the heart. Contrary to digitalis, in physiological doses it is, with regard to the heart, a tonic and hyposthenic. In physiological doses it is not spoliative as digitalis. Continuing its use has not, then, the same dangers. Its use seems to be as an indirect compensator. In super-exciting the sensibility and contractibility of animal life, it causes morbid activity of the nervous system and the contractile fibres of vegetable life. Its action is very distinct from that of digitalis. When the latter is ineffectual, the former may be appealed to as well at digitalis. Veratrine is contraindicated in the last period of cardio-vascular affections, and in asystole. Finally, there is ground for trying it in all the diseases which affect the nervous system and vegetable life. *Med. and Surg. Reporter.*

---

**Diabetes.**

In the *Wiener Med. Wochenschrift*, Dr. Bischoff relates the case of a patient, forty-four years old, who had for thirty-two days discharged on an average daily 2,885 grammes of urine and 25.6 grammes of sugar. In consequence of intercurrent occipital neuralgia, galvanism was applied to the neck. The negative electrode was applied high on the neck, and the positive between the angle of the lower jaw and the mastoid process, then over the supra-spinous fossa ; and again, the positive pole being retained in the latter position, the negative pole was drawn twelve or fourteen times over the course of the occipital nerve and the cervical spinous processes ; finally, the negative pole was applied alternately in the right and left supra-clavicular fossæ, and the positive in the epigastrium and over the liver. This treatment was continued, the application in each place being made for one or two minutes daily, for thirty-two days.

The daily quantity of urine became reduced to 2,056 grammes, and that of sugar to 11.69 grammes. Two months after the galvanism had been left off, an examination of the urine for fourteen days showed that the quantity discharged daily had risen to 2,488 grammes, and that of sugar to 19.87 grammes. On the death of the patient, a year and a half afterwards, there was found to be chronic disease of the vessels in the floor of the fourth ventricle and the neighboring parts of the brain. —*Phila Med. and Surg. Jour.*

---

**Baldness.**

A writer to an English exchange gives the following case, which will be read with interest by that large class of gentlemen whose hair is thin, but not from years :

A gentleman who had lost nearly all his hair after a very severe attack of fever, consulted a French physician of great reputed success as a hair restorer. The prescription was a drachm of the homœopathic tincture of phosphorus to one ounce of castor oil, the bare spot to be rubbed with this mixture three times weekly for half an hour each time, after the skin of the head had been thoroughly cleansed with warm water without soap. This treatment was faithfully carried out for about six months ; the hair soon began to grow, and in a year from the time of first following the doctor's advice, his head was as thoroughly covered as ever, the new crop of hair being about two shades darker than the old.—*Med. and Surg. Reporter.*

---

**Intermittent Fever.**

It is allowable for the patient, after admission to the Roosevelt Hospital, N. Y., to have one chill, in order to determine the character of his disease ; but subsequent to that the recurrence of chills are not permitted. The certain arrest of the disease is looked for under the influence of what are denominated "Clark's powders," which are formulated as follows :

℞

Quiniæ sulph., grs. x ;
Pulv. capsicum, grs. iij ;
Pulv. opii. gr. j.

The patient receives one powder twelve hours previous to the time for the recurrence of the chill, and another about one hour previous. The powders are continued in this manner for three or four days, and then continued every morning as long as may be desired. It not infrequently happens that the chills return after they have been stopped, and the most common time for them to return is at the end of two weeks. Near the expiration of two weeks, therefore, it is well to return to the full sized powders, if they have been diminished in size, and continue them in full doses, for three or four days, twice a day, as at the outset. In cases which have been of long standing and have proved themselves very entractable, when the chills have been once stopped, the remedy should be continued over three or four periods of return at least. By this method of treatment cases of chills and fever can be cured which have revisited all former treatment.

---

**Prevention of Loss of Blood.**

The *Medical Times & Gazette* recommends the following plan to diminish the loss of blood in operations :

An elastic bandage about two inches and a half in width, and from five to ten yards long is firmly bound around the limb, commencing at the toes or fingers, as the case may be, and is then continued upwards so as to drive the blood before it out of the veins and arteries, When the desired point has been reached, a strong india rubber band about half an inch in diameter is tightly drawn two or three times around the limb, just above the elastic bandage and fastened by hooks. The bandage is then removed, leaving the tissues blanched and exsanguined. Not a particle of blood is lost during the operation, which is really more bloodless than when performed on the dead subject. After the operation is completed, the rubber rope is removed and the blood then finds its way into the vessels, which are ligatured or twisted according to the taste or inclination of the surgeon. In this plan, which has been carried out at St. Thomas', Guy's, London, and St. Bartholomew's Hospitals, many operations have now been performed, including excision of the knee and elbow joints, amputations, and the removal of dead bone ; and Mr. Wagstaffe has recently amputated through the thigh for gangrene of the foot on this plan, the precaution having been taken to commence the application of the elastic bandage several inches above the mortified part. No ill effects of any kind have hitherto been observed from the use of this contrivance, although the duration of the operations has varied from a few minutes up to half an hour, and even more, during the whole of which time the circulation has been completely arrested, no evidence has been found of the formation of emboli or thrombi in any of the cases. But it is one of the possible evils of the device that the prolonged pressure on the vessels and complete stoppage of circulation may, under certain conditions, lead to the formation of a clot, which, on the establishment of circulation, may be carried along the vessels, and arrested in some part of their course, giving rise to circumscribed inflammations or even gangrene. There is also considerable danger in applying the bandage over parts which are inflamed and suppurating, especially if decomposition be going on, lest some of the clots which are found in the blood vessels of the affected parts be detached and forced into the blood current. For such cases it would be well to employ in addition a modification of the plan which has been practiced at Edinburgh for the last two or three years, which consists in suspending the limb for some minutes before the operation, so that the blood may gravitate downwards. Then the bandage may be applied at the proximal side of this diseased part, thus avoiding all risks of the septic poisoning or embolism.

---

**Bromide of Sodium as a Nervous Sedative.**

Dr. Ainslee Hollis, in the London *Practitioner*, says that the bromide of sodium, although known to have a therapeutic action analogous to the bromide of potassium, has lately somewhat fallen into disuse, while the latter salt has been recommended as a remedy for nearly every disease. The bromide of sodium has a pungent saline taste, is freely soluble in water, and forms a colorless solution. Shortly after it is taken into the stomach, a burning sensation is experienced at the epigastrium ; this quickly passes off, giving place to drowsiness and sleep, followed by numbness in the extremities, which does not disappear until several hours after waking.

The following cases illustrate its beneficial action in nervous diseases : Fred. B., æt. 21 years, came under treatment for epilepsy, November 13, 1872. From early childhood the attacks had occurred three or four times weekly. He was treated for the first month with thirty-grain doses of bromide of potassium thrice daily, combined with one-third of a grain of extract of belladonna at night. As the attacks diminished but little in frequency, a seton was inserted in the back of his neck ; for a short time after this the fits appeared to be less frequent, and severe, but, as he complained of lowness of spirits and debility, one grain each of sulphate of iron and sulphate of zinc were ordered to be taken three times daily. This was continued only one week, as the symptoms returned with their former severity. Bromide of potassium was resumed in forty-grain doses ; this, with the addition of succus conii and a fresh seton, composed the treatment up to May 22, 1873, no effect being produced. On the 22d he was ordered three grains of bromide of sodium three times a day ; and during the next week he had only two fits. On May 29, each dose was increased to fifteen grains, and on June 5, to twenty grains ; in the interval he had several attacks of "petit mal," but no marked epileptic seizures. After this he improved much in general health, and had but one fit. In another case, a boy 14 years old, who had been subject to epilepsy from birth, after subduing the paroxysms by taking ten grains of bromide of potassium three times daily, suffered no return on the substitution of three-grain doses; here, also, general depression was marked. This depression of the spirits very frequently accompanies the use of the medicine in a watery solution, and might possibly be counteracted by the administration of a tonic in combination.

In two cases of nervous excitement, due to mental anxiety, and in one of epileptic vertigo, small doses of the bromide produced great relief, while in a case of insomnia, in an old man, it appeared to do harm. We have, therefore, in sodic bromide, where judiciously used, a valuable nervous sedative.—*Med. Times.*

